# Strengthening intellectually challenged adolescents’ sense of self: An appreciative inquiry mixed-methods intervention

**DOI:** 10.4102/hsag.v23i0.1113

**Published:** 2018-11-22

**Authors:** Christene J. Louw, Hermanus B. Grobler, Richard G. Cowden

**Affiliations:** 1 Community Psychosocial Research (COMPRES), North-West University, South Africa

## Abstract

**Background:**

Individuals with intellectual challenges may experience a sense of isolation within their families. How their families and friends react towards these challenges influences the formation of their identity and self-acceptance significantly.

**Aim:**

The aim of this research was to explore and describe how the sense of self of intellectually challenged adolescents could be strengthened within familial relationships and to evaluate the effectiveness of appreciative inquiry as an intervention approach.

**Setting:**

The study was conducted with families at a school for children with intellectual disabilities in the Ekurhuleni Metropolitan Area, Northern Region of Gauteng, South Africa.

**Methods:**

In an embedded mixed-method approach, a sample of 24 intellectually challenged adolescents and their families were selected, tested and interviewed. Quantitative data were collected using the BarOn Emotional Quotient Inventory (BarOn EQ-i:YV) on adolescents and the Family Environmental Scale (FES) on families in the experimental and control groups, before and after intervention. Qualitative data were gathered through an appreciative inquiry intervention and semi-structured interviews with adolescents in the experimental group.

**Results:**

Although the quantitative findings were not statistically significant, the qualitative findings indicated that adolescents and family members experienced the sense of self of intellectually challenged adolescents positively, rather than as ‘disabled’. The appreciative inquiry shows potential to strengthen intellectually challenged adolescents’ sense of self in a supportive, positive family environment.

**Conclusion:**

The research is valuable in the way it highlights the importance of relational research in cases where quantitative research does not seem to be effective.

## Introduction and background

According to Baron and Byrne (2000) and Anderson and Sabatelli (2011), the family is a living system, a unit of interdependent relationships, which plays a crucial role in the formation of the self, or identity (cf. Amoateng et al. 2004; Becvar & Becvar 2013). It is within their families that people develop and construct an identity, discover who they are, form close relationships and receive support and encouragement (Fomby & Osborn 2010; Silkos & Kerns 2006). Polster and Polster (1973) see this discovering as a becoming aware of, whereas Cottle (2003) regards it as a process of affirmation or being strengthened. This in turn leads to the emergence of an autonomous sense of self (Pietromonaco & Feldman Barrat 2000).

In this research study, intellectually challenged adolescents are not considered in isolation but are seen as part of a group – a dynamic familial system. They too are searching for answers about their world, their identities, their abilities and about themselves. This exploration to find answers is characteristic of adolescence (Marcia 1980). The importance of the family system is supported by appreciative inquiry, which is the study of what gives life to human systems when they function at their best (Cooperrider, Whitney & Stavros 2008). Appreciative inquiry was therefore seen as a good fit as a potential method to strengthen the sense of self of intellectually challenged adolescents. According to Watkins and Stavros (2010), appreciative inquiry suggests that human organisation and change at its best is a relational process of inquiry, grounded in affirmation and appreciation (Whitney & Trosten-Bloom 2010). The art of ‘appreciation’ is the art of discovering and valuing factors that give ‘life’ to a living system (Cooperrider et al. 2008). Appreciative inquiry originated as a theory and as a qualitative research technique with five core principles, namely the constructionist, simultaneity, poetic, anticipatory, positive and wholeness principles (Cooperrider et al. 2008; Gergen 1995). Whitney and Trosten-Bloom (2010) later added two additional principles, namely enactment and free choice. This innovative approach creates new possibilities and meaningful changes in all living systems by means of a ‘5-D’ cycle process: define, discover, dream, design and destiny (Watkins & Stavros 2010). The process involves the art and practice of asking positive questions, interviewing and storytelling in order to draw out the best of the past, to understand what one wants more of and effective visualisation of the future (Cooperrider et al. 2008; Watkins & Stavros 2010). It is from this stance that appreciative inquiry as an intervention approach was selected and applied during family meetings in this study.

### Problem statement

Goodley and Tregaskis (2006) point out that people with developmental disabilities are aware of their difference and exclusion from society, which they perceive as stigmatisation attached to the disability. This stigmatisation influences the formation of their social identity and interaction with others negatively (Gwernan-Jones 2008; Michailakis 2003). Davis and Gavidia-Payne (2009) are of the opinion that stigmatisation and lack of support from families and friends can intensify feelings of inadequacy, frustration and rejection in people with intellectual disabilities, which influence how they view themselves (cf. Canary 2008; Gill 2001; Hughes, Russell & Paterson 2005). However, a study by Schwartz and Gidron (2002) found that the formation of the self in disabled individuals depended more on the relationships the disabled have with their families and society than on the stigma attached to their disabilities (cf. Upadhyaya & Havalappanavar 2008). Thus far, the literature has provided limited insight into intellectually challenged adolescents’ sense of self and the influences of familial relationships on the configuration and affirmation of the self. Prior research on intellectually challenged adolescents and their relationships has focused predominantly on traditional problem-solving processes, which, according to Koepke and Denissen (2012), separate and dissect pieces of the system, overlooking important factors such as the evolving experiences of familial relationships, whether these are positive or negative (Nachshen, Woodford & Minnes 2003; Woodbridge, Buys & Miller 2011).

### Aims of the study

The aims of this research study were to explore and describe how the sense of self of intellectually challenged adolescents could be strengthened within familial relationships and to evaluate the effectiveness of appreciative inquiry as an intervention approach.

### Definition of key concepts

#### Intellectually challenged adolescents

In this study, the term ‘intellectual and developmental disabilities’ refers to a general, broad concept, whereas the term ‘intellectually challenged’ has been chosen to refer to the specific adolescents in this study. Intellectual challenge or disability is a neuro-developmental disorder that occurs before the age of 18 and is characterised by an intelligence quotient (IQ) of 70 or below (The American Association of Intellectual and Developmental Disabilities 2013). In terms of the American Psychiatric Association’s (APA) *Diagnostic and Statistical Manual of Mental Disorders* classification, the level of disability is divided into five categories based on IQ: *mild, moderate, severe, profound* and *unable to classify* (APA 2013). Significant limitations are present in two main areas: intellectual functioning and adaptive behaviour (APA 2013).

#### The self

The self is seen as a complex psychological structure; from a gestalt theory perspective, it is seen as a system of contacts (Yontef 1993), a process that is continuously becoming and evolving (Perls, Hefferline & Goodman 1951). According to Gergen (2011) the self is continuously constructed through socially created language and conversations within relationships, belonging to a group (family), being valued and accepted by that group. This in turn leads to a social identity (cf. Cooperrider Dole et al. 2008; Stets & Burke 2000).

## Research methodology

### Research design

The procedure involved an embedded mixed-method experimental design (Creswell & Plano Clark 2011), which included quantitative and qualitative data collection and analysis (Delport & Fouché 2011). The quantitative data in this study were gathered by means of standardised tests, the BarOn Emotional Quotient Inventory: Youth Version (BarOn EQ-i:YV) developed by Bar-On and Parker (2000) and the Family Environmental Scale (FES) developed by Moos and Moos (2009). The use of these instruments was embedded within the qualitative data collection by means of the appreciative inquiry intervention and semi-structured interviews with the participating adolescents and their families.

### Research process

#### Population and sampling

A pilot study was conducted by selecting one intellectually challenged adolescent and his or her family, randomly and purposefully, from the population. A pilot study according to Strydom and Delport (2011) gave direction to the main investigation by determining the feasibility of the study, reviewing the literature, engaging in discussions with experts in the field, testing the measuring instrument and identifying possible problems that might arise, in order to modify or streamline the main research. This adolescent and his or her family did not form part of the final sample.

A sample of 24 intellectually challenged adolescents with mild (50–75) to moderate (35–55) intelligence disability, between the ages of 11 and 14 years, were selected for the study.

Family members living in the same household as the intellectually challenged adolescents formed part of the study. The study was conducted at a school for children with intellectual disabilities in the Ekurhuleni Metropolitan Area, Northern Region of Gauteng, South Africa.

Two introductory meetings were held with the selected intellectually challenged adolescents and their families to explain the purpose and nature of the study before commencement of tests and the intervention.

The families represented different family types (see Table 1). They were randomly divided into an experimental group and a control group.

**TABLE 1:** Sample characteristics.

Variable	Intervention group			Control group			Total		
	*N*	*M*	*SD*	*N*	*M*	*SD*	*N*	*M*	*SD*
**Adolescents**									
**Age**	12	12.17	0.94	12.00	12.25	1.22	24.00	12.21	1.06
**Gender**									
Male	8	12.13	0.83	8.00	12.63	1.30	16.00	12.38	1.09
Female	4	12.25	1.26	4.00	11.50	0.58	8.00	11.88	0.99
**Race**									
Black	5	12.00	1.00	3.00	12.50	1.29	8.00	12.22	1.09
White	7	12.29	0.95	9.00	12.13	1.25	16.00	12.20	1.08
**Adolescents’ families**									
**Family structure**									
Intact	6	30.57	14.23	5	29.74	18.73	11	30.14	16.55
Single-parent	3	32.00	12.81	5	25.71	12.89	8	27.37	12.83
Multigenerational	1	40.40	19.78	-	-	-	1	40.40	19.78
Skip-generational	1	49.50	3.54	-	-	-	1	49.50	3.54
Divorced or extended	1	29.86	15.64	2	30.83	20.15	3	30.31	17.08
***N***	12	-	-	12	-	-	24	-	-
Parents	19	42.11	6.97	19	39.74	6.60	38	40.92	6.80
Grandparents	4	53.75	5.56	3	61.33	4.16	7	57.00	6.14
Siblings	17	17.47	5.01	21	13.81	7.41	38	15.45	6.63
***n***	40	-	-	43	-	-	83	-	-

*n*, frequency, *N*, total frequency, *M*, mean, *SD*, standard deviation.

#### Data gathering

The data collection process took place from February 2014 to July 2014. The BarOn EQ-i:YV *pre-intervention* and *post-intervention tests* were administered to the 24 intellectually challenged adolescents in the experimental and control groups at the school. The FES questionnaires were sent via an existing communication system at the school to family members to be completed by both the experimental and control groups’ families. Thereafter, 12 appreciative inquiry intervention meetings with the experimental group’s family members took place on Saturdays, to accommodate family members who worked on weekdays. Each appreciative inquiry intervention took approximately 2–6 hours, depending on the size of the family unit. The *discovery* and *dreaming* phases were grouped together, as were the *designing* and *destiny* phases, in order to ensure continuation and integration. After the completion of the family intervention sessions, semi-structured interviews were held with the intellectually challenged adolescents from the experimental group.

#### Quantitative measuring instruments

The BarOn EQ-i:YV (Bar-On & Parker 2000) was chosen to measure the emotional and social intelligence of the participating adolescents. As there is no instrument that measures sense of self as a construct, the decision was made to use the BarOn EQ-i:YV because it measures certain components of a sense of self on the intrapersonal and interpersonal scales, which are closely linked to self-awareness, self-perceptions, feelings and relationships with others. The instrument consists of 60 Likert-type items, anchored at 1 (*very seldom* or *not true of me*) and 4 (*very often true* or *true of me*), and divided into eight subscales, of which four subscales combined measured the total emotional intelligence: (1) intrapersonal (six items – e.g. ‘I can easily describe my feelings’), (2) interpersonal (12 items – e.g. ‘I can tell when one of my close friends is unhappy’), (3) stress management (12 items – e.g. ‘I can stay calm when I am upset’), (4) adaptability (10 items – e.g. ‘Even if things get hard, I do not give up’). The factor structure of the BarOn EQ-i:YV has been found to be valid during the initial development (Bar-On & Parker 2000) and this finding was supported in subsequent studies (Parker et al. 2005). The instrument is able to differentiate between primary and high school students at various levels of academic achievement in terms of grade point averages, as higher scores are recorded for students clustered into more successful groups (Eastabrook, Duncan & Eldridge 2005, cited in Parker et al. 2009). The BarOn EQ-i:YV scales’ internal consistency has been found to be satisfactory to strong (*a* = 0.65–0.90) across several studies (Harrod & Sheer 2005; Parker et al. 2008). Acceptable test–retest reliability has also been found at an interval of 3 weeks for the Emotional Quotient (EQ) scales (*r* = 0.70–0.89) and for the General Impression and Positive Impression and General Mood scales (*r* = 0.60–0.77) (Hassan & Sader 2005).

The FES (Moos & Moos 1994, 2009) is a social climate instrument that is used to assess family environment from different members’ perspectives. The instrument contains 10 subscales, consisting of 90 true–false items (1 = false, 2 = true) that focus on the dimensions of personal growth, relationship and system maintenance. The relationship dimension was of special interest to this study. It is comprised of the subscales *cohesion* (nine items – e.g. ‘There is a feeling of togetherness in our family’), *expression* (nine items – e.g. ‘We tell each other about our personal problems’) and *conflict* (nine items – e.g. ‘Family members often criticise each other’). The participants were asked to complete all the items according to their actual (real) family experiences. The FES’s internal consistency has been found to be in an acceptable range for all 10 subscales: for the relationship dimension relevant to this study, the internal consistency for *cohesion* was 0.78, for *expression* 0.69 and for *conflict* 0.75 (Moos & Moos 1994, 2009). The test–retest reliability on the subscales for Form R (actual and real) is in an acceptable range and varies from a low of 0.68 to a high of 0.86 on the 2-month test–retest, to relatively high for the 4-month interval (Moos & Moos 1994, 2009).

#### Qualitative intervention process

The process is summarised in Table 2.

**TABLE 2:** Appreciative inquiry ‘5-D’ intervention process that underlined relevant principles.

Phases	Relevant questions	Principles
Defining phase	*Identifying the topic of inquiry* The research topic was identified in working with the intellectually challenged adolescents and their families.	*Poetic principle*: We can choose what we study, to create, influence and make a difference in the world. *Constructionist principle*: Words create worlds (Gergen, Gergen & Schrader 2009). Identity continuously and socially created through language and conversations (Gergen 2011).
Discovery phase	*Valuing and appreciating ‘what is’* Questions were asked about families’ best experiences, individually and collectively: Tell me about a time, an event or experience that you had within your family, when you were really happy or excited to be part of your family? Describe the event in detail: What was happening? Who was involved? What did you do? What made it such an exciting or enjoyable experience? How did you and your family feel at the time? (These questions supported the FES questions: *expression* and *cohesion*). *Core life-giving factor or values*: What do you deeply value and appreciate about your family and yourself? And what do you think your family values most about you? What is the one thing or gift that makes your family unique, the way it is right now? (These questions supported the BarOn-i:YV intra- and interpersonal scales). *Facing conflict and challenges*: Tell me about an event when you experienced conflict within your family. How did you resolve it and what have you learnt from it? (These questions supported the FES questions: *conflict*).	*Simultaneity principle*: Inquiry is an intervention and creates change. Questions stimulate ideas, innovation. *Positive principle*: Positive change is enabled through discovery and by mapping the positive core. Positive expression grows possibilities. *Wholeness principle*: Collective creativity brings out the best in people. The ‘I’ becomes a ‘we’.
Dreaming phase	*Envisioning and imagining ‘what might be*’ If you had three wishes for your family, what would they be? What is happening and what is different within your family while dreaming?	*Anticipatory principle*: Images inspire actions, and create conversations and inner dialogue. Human systems move in the direction of their images of the future (Gergen & Gergen 2004).
Designing phase	Dialoguing and determining ‘what should be’ This is a planning phase where wishes become a reality by crafting provocative propositions, and statements are written in the present tense: What small steps or changes can you and your family (individually and collectively) take to accomplish or achieve these dreams?	*Enactment principle*: Acting ‘as if’ is self-fulfilling. Positive change occurs when the process used to create the change is a living model of the ideal future.
Destiny phase	*Innovating and creating ‘what will be*’ It is a visual or concrete representation on the path forward, giving direction and commitment to what needs to be done – to support and strengthen relationships. The following questions were asked: What have you learnt from this experience? What were the highlights of this family intervention? How can you apply this in your daily interactions with your family members going forward? Do you see your family members differently?	*Free-choice principle*: Free choice liberates power. People are more committed when they have freedom to choose how and what they contribute towards.

**Semi-structured interviews**

Semi-structured interviews were held with the intellectually challenged adolescents in the experimental group, focusing on how they experienced their sense of self, as well as how they experienced their relationship with family members.

Typical questions were:

- Tell me about how you see yourself within your family.
- Is there anything that you want to change about yourself? If so, what would that be?
- Tell me about how you experienced your family ‘meeting’ (intervention).
- How was the experience for you?
- Can you recall the ‘meeting’, looking at your family photos and your family drawing?

As suggested by Milewski-Hertlein (2001), genograms were used to evoke memories of the intervention and the stories shared, and photographs were taken during the family meeting as a shared activity.

Typical questions were:

- What did you enjoy about the family ‘meeting’ and how did it make you feel?
- What were the things that your family talked about that you did not know about yourself?
- After the ‘meeting’ (intervention), what is different about your family and how you think and feel about yourself?
- What has changed?

#### Data analysis

The *qualitative data analysis* involved identifying patterns, constructing a framework – according to Babbie (2007) and Creswell (2007) a framework can communicate the essence of the data revealed – and discovering underlying meanings and relationship patterns. A six-phase thematic method developed by Braun and Clarke (2006) was selected to analyse and describe the textual and visual data. Initial codes and themes were identified from the raw data and verified.

*The quantitative analysis.* Raw scores on the relevant BarOn EQ-i:YV and FES scales were converted to standardised scores, and descriptive statistics were computed for each of the relevant scales. The primary purpose was to examine changes in the scores prior to and after the intervention. Intervention and control group, 2 × 2 (group × time) mixed analyses of variance (ANOVAs) were performed on the relevant scales as dependent variables to determine the main interaction effects (see Table 3). Before proceeding with parametric testing, all hypothesis testing assumptions were tested and appropriately fulfilled. An alpha level of 0.05 was used for all statistical analyses. Partial eta-squared () values were computed as estimates of effect size, which were evaluated using Cohen’s (1992) effect size standards of 0.02 (small), 0.12 (medium) and 0.28 (large).

**TABLE 3:** Descriptive statistics for dependent variables across experimental conditions from Time 1 to Time 2.

Dependent variables	Intervention group				Control group			
	Pre-intervention test		Post-intervention test		Pre-intervention test		Post-intervention test	
	*M*	*SD*	*M*	*SD*	*M*	*SD*	*M*	*SD*
Intrapersonal	103.75	12.1	108.33	11.16	99.08	10.88	103.67	11.87
Interpersonal	93.67	12.1	100.00	13.48	103.42	15.11	100.67	20.07
General mood	104.5	7.23	108.5	9.25	107.5	10.18	103.83	14.58
Family Cohesion	38.17	8.85	47.58	3.89	42.08	5.84	41.92	7.54
Family expression	52.42	6.67	57.25	5.03	50.5	9.56	52.08	10.08
Family conflict	57.25	4.67	57.83	5.44	58.83	4.84	56.42	5.25

*M*, mean, *SD*, standard deviation.

### Ethical considerations

Ethical clearance was obtained from the North-West University (NWU-00060-12-A1), Potchefstroom, in South Africa, and permission was granted by the South African Department of Education to conduct the research in the relevant school. Informed consent was obtained from parents and legal guardians, and written assent from the intellectually challenged adolescents and siblings. Participants were assured that their participation was voluntary and that they could withdraw from the study if they wished; it was explained that no remuneration was offered and that the information they gave would be kept confidential and anonymous.

## Results

### Quantitative results

The pre- and post-intervention test descriptive statistics for both the experimental and the control groups are reported in Table 3. For the BarOn EQ-i:YV Intrapersonal scale, the 2 × 2 (group × time) mixed ANOVA revealed the group by time interaction effect, *F* (1, 22) = 0.00, *p* = 1.000, $\eta_{p}^{2}$ = 0.000; the main effect for condition, *F* (1, 22) = 1.28, *p* = 0.270, $\eta_{p}^{2}$ = 0.055, was not statistically significant. Although the main effect for time was not statistically significant, *F* (1, 22) = 3.83, *p* = 0.063, $\eta_{p}^{2}$ = 0.148, a medium effect size was found. Therefore, it seems that both groups’ *intrapersonal skills* improved in the pre- to post-intervention test interval, but the improvements tended to be similar across both groups. Similar results were found for the 2 × 2 mixed ANOVA on the *interpersonal scale*: the interaction, *F* (1, 22) = 2.09, *p* = 0.163, $\eta_{p}^{2}$ = 0.087, the main effect for time, *F* (1, 22) = 0.33, *p* = 0.574, $\eta_{p}^{2}$ = 0.015, and the main effect for group, *F* (1, 22) = 0.90, *p* = 0.353, $\eta_{p}^{2}$ = 0.039, were not statistically significant. Considering the small to medium effect size found for the interaction and the descriptive statistics in Table 2, it seemed that the experimental (intervention) group improved from Time 1 to Time 2, whereas the control group displayed a reduction in *interpersonal skills* over time.

On the *General Mood* scale, the interaction effect for the 2 × 2 (group × time) ANOVA was marginally non-significant, *F* (1, 22) = 3.27, *p* = 0.084, $\eta_{p}^{2}$ = 0.129, although this result was medium in effect size (Cohen 1988). The pattern of group scores suggests that the experimental (intervention) group’s general mood improved, yet appeared to decline among the control group from Time 1 to Time 2. Neither the main effect for time, *F* (1, 22) = 0.01, *p* = 0.938, $\eta_{p}^{2}$ = 0.000, nor group, *F* (1, 22) = 0.048, *p* = 0.828, $\eta_{p}^{2}$ = 0.002, was statistically significant.

The 2 × 2 (group × time) mixed ANOVA for the *FES scale of cohesion* demonstrated a significant group by time interaction, *F* (1, 22) = 12.47, *p* = 0.002, $\eta_{p}^{2}$ = 0.362, and main effect, *F* (1, 22) = 11.61, *p* = 0.003, $\eta_{p}^{2}$ = 0.345. The large effect sizes support greater increases in family cohesion among the intervention group from the pre-intervention test to the post-intervention test, as compared to the scores of the control group. However, the main effect for group was not statistically significant, *F* (1, 22) = 0.13, *p* = 0.721, $\eta_{p}^{2}$ = 0.006.

For the *Expression* scale, the group by time interaction was not statistically significant, *F* (1, 22) = 1.06, *p* = 0.315, $\eta_{p}^{2}$ = 0.046. Although the main effects for group, *F* (1, 22) = 1.49, *p* = 0.236, $\eta_{p}^{2}$ = 0.063, and time, *F* (1, 22) = 4.12, *p* = 0.055, $\eta_{p}^{2}$ = 0.158, also did not reveal any significant differences, the time main effect was medium in effect size. This suggests that families’ levels of expression increased in a similar way from the pre-intervention test to the post-intervention test across both groups.

The 2 × 2 (group × time) mixed ANOVA for the *Conflict* scale indicated that the interaction effect, *F* (1, 22) = 1.81, *p* = 0.193, $\eta_{p}^{2}$ = 0.076, the main effect for time, *F* (1, 22) = 0.674, *p* = 0.420, $\eta_{p}^{2}$ = 0.030, and the main effect for group, *F* (1, 22) = 0.002, *p* = 0.962, $\eta_{p}^{2}$ = 0.000, were not statistically significant. Neither group seemed to evidence statistically meaningful changes in family conflict from Time 1 to Time 2.

### Qualitative results

Themes that were identified during the appreciative inquiry family meetings, and those from the semi-structured interviews with the intellectually challenged adolescents from the experimental group, were compared with the test results obtained from the BarOn EQ-i:YV and the FES, *pre-intervention* and *post-intervention.* The results obtained in the construct *cohesion, expression* and *conflict* and results of the interpersonal, intrapersonal and general mood were linked together, to determine any statistical significant changes. These results were put in context with the themes identified during the appreciative inquiry process, *discovery, dreaming, designing* and *destiny*, to determine whether the method was successful in strengthening adolescents’ self-perception, self-awareness and family relations. The data collection process took place by administering the quantitative standardised pre-tests, followed by the qualitative intervention process, the quantitative post-tests and the semi-structured qualitative interviews with adolescents. The study was a mixed-method study where the quantitative design was imbedded in the qualitative research design and process. See a joint display of the quantitative and qualitative findings for the experimental group in Table 4.

**TABLE 4a:** Joint display of quantitative and qualitative findings for the experimental group.

Variables	Appreciative inquiry intervention themes	Semi-structured interview themes (after intervention)
**Family Environmental Scale (FES)** **Family cohesion** *Pre-intervention test*: 38.17 (8.85) *Post-intervention test*: 47.58 (3.89) *large effect size supports greater increase in cohesion.	*Family experiences*: Close bonds and connections, togetherness, belonging to, part of (inclusion), fun and excitement; love and acceptance, nurturance and supportiveness. ‘We are a normal family – just like others: socialising, disciplining and parenting.’ (F1, female, 11 years old) ‘Families cares for each other.’ (F6, male, 11 years old)	*Family time*: Togetherness, fun, family memories. Appreciative inquiry meeting: taking photographs and drawing genograms created fun and togetherness. Majority of adolescents expressed the desire to have more quality time with their family members.
**Family expression** *Pre-intervention test*: 52.42 (6.67) Post-intervention test: 47.58 (3.89) *medium effect size changes in expression	*Family strengths and core values*: Family roots, religious and cultural beliefs, unconditional love, positive attitudes, embracing challenges. Creating safe and secure family environment. Expressed high levels of responsibility, protection, especially from grandparents and siblings. ‘Family prays together.’ (F10, male, 13 years old) ‘We come from a proud family. Our culture is important to us.’ (F4, female, 12 years old)	*Family relationships*: ‘Family means everything, I am nothing without my family.’ (A3, female, 14 years old) ‘I want to feel that I belong to a family. I want to make them feel proud of me.’ (A4, female, 12 years old) Adolescents not focused on disability but on close relationship with parents and siblings.
**Family conflict** *Pre-intervention test*: 57.25 (4.67) *Post-intervention test*: 57.83 (5.44) *no statistically significant changes	*Family challenges*: Addressed feelings of adolescents and siblings about not being valued, respected, accepted and included in family decisions. Family liked to be seen as a ‘normal’ family, without judgement. ‘We focus on limitations and challenges – not disabilities, not on normal or abnormal.’ (F1, female 11 years old) ‘We do not see our family as different from other families. We try to create a sense of belonging and connection with the larger community – through function and activities.’ (F2, female, 12 years old)	*Adolescents’ challenges*: Seek independence, acceptance, to be heard. Afraid not meeting expectations and to be judged. Blaming and name calling – increased feelings of rejection. ‘I feel it is my fault. My parents are ashamed of me.’ (A10, male, 13 years old) ‘I always hear about the things that I cannot do and not what I can do.’ (A6, male, 11 years old)

**TABLE 4b:** Joint display of quantitative and qualitative findings for the experimental group.

Variables	Familial experiences on sense of self	Adolescents’ experience of sense of self
**BarOn EQ-i:YV:** Intrapersonal *Pre-intervention test*: 103.75 (12.51) *Post-intervention test*: 108.33 (11.16) *a medium effect size, suggests an improvement.	Majority of family members described positive and warm feelings towards the adolescent. Proud of accomplishments and supportive – based on strong family values and religious beliefs. ‘Our grandson is a gift from God.’ (F11, male, 12 years old) ‘We are trying to make sense of this disability by focusing on the spiritual.’ (F5, male, 13 years old) Siblings and parents or grandparents more focused on forming a relationship with the adolescent. Some family members questioned own their own sense of self, their parenting skills and capabilities to take care of and help adolescent develop to full potential. Intrapersonal feelings of guilt and shame were expressed by some family members (because of rejection by society and own family). ‘My family and community rejected me because of my disabled son. To be disabled is seen as a curse.’ (F10, male, 13 years old)	Described different selves: connected to positive and negative experiences, characteristics attached to abilities and inabilities (seen as disabilities). Referred to gender, gender roles and physical traits. Abilities were seen as capable selves and accepted by society. Disabilities (visible and unable to perform) were seen as disabled selves and rejected by society. ‘I cannot do what other boys do – normal things like sports.’ (A6, male, 11 years old) ‘My mother is ashamed of me.’ (A10, male, 13 years old) ‘My father left us because of me – I am slow. My friends call me a retard.’ (A9, male, 11 years old) Strong religious and cultural selves. ‘I am a child of God, created by God.’ (A2, female, 12 years old) ‘I am a Zulu, Italian, Sepedi or Tswana-speaking boy/girl and named after my grandfather/mother.’ (A3,female, 14 years old; A4, female, 12 years old; A5, male, 13 years old and A6, male, 11 years old) Majority of adolescents’ experiences on sense of self were positive and embedded within relationships.
**Interpersonal** Pre-intervention test: 93.67 (12.10). *Post-intervention test*: 100 (13.48) *small to medium effect size suggests improvement	*Expressed endearing characteristics/ attributes of adolescents*: Being lovable, kind-hearted, free-spirited seen as self. For the majority of family members, the relationship was more important than the disability. *Positive and negative experiences*: Based on acceptance of society and capabilities of the adolescent. Proud of the adolescent’s achievements. Protective towards the adolescent to evade judgement and rejection from peers and society. Most families had a positive outlook on life – they embrace challenges, focus on mastering of skills and on the independence of the adolescent. Most of the siblings had a special relationship or bond with the intellectually challenged adolescent. ‘She is my best friend.’ (A1, female, 11 years old). ‘My sister is so funny, she makes us laugh. She keeps us as a family together.’ (A3, female, 14 years old).	*Family relationships*: Vitally important for the formation of the self; most of the adolescents expressed a desire to have closer and more affectional bonds with their family members. They seek acceptance from their family, and this is more important than their disability. *Relationships with peers, friends*: The adolescents experienced difficulties in forming and maintaining relationships (disability hinders contact-making and acceptance). Adolescents compared themselves to typically developed adolescents without disabilities. Most felt rejected by their social group and the larger community. ‘I am aware of my disabilities and ashamed to be in a school for disabled children. I am not normal.’ (A6, male, 11 years old; A7, male, 13 years old). ‘It is hard for me – I do not fit in and felt dumb.’ (A5, male, 13 years old).
**General mood** *Pre-intervention test*: 104.50 (7.23) *Post-intervention test*: 108.50 (9.25) *medium effect size suggests improvement	*During appreciative inquiry*: Families were open in sharing their feelings and experiences from a positive stance. This created a mood of light-heartedness, jokes and fun, which was enhanced by taking family photos and togetherness.	*During appreciative inquiry*: Participants were initially anxious. General mood changed to positive because of fun and togetherness, positive comments made by family members, which had a positive impact on how adolescents viewed themselves. *During semi-interviews*: Narratives revealed that the majority of adolescents felt that they need to perform ‘better’ in order to get love and acceptance from parents.

After the completion of the research process, two follow-up sessions with the control group took place at the school: one where feedback was given to adolescents and their families regarding the quantitative test results and secondly a joint appreciative inquiry process meeting with families.

## Discussion

During the *discovery* phase, family members selected stories to describe their best experiences of being part of the family individually and collectively. During this phase families discovered the best of what had been and was then currently being practised (Cooperrider et al. 2008). Families’ narratives referred to special occasions, such as birthdays, holidays, family and cultural gatherings, where the whole family was present (including grandparents, uncles, aunts and cousins). Families described events surrounding the birth of the intellectually challenged adolescent, growing up and openly expressed love and care. Most of the intellectually challenged adolescents and some family members voiced their surprise at the stories surrounding them.

Responses from families (F) and adolescents (A) are cited verbatim and printed in italics to give feedback on the appreciative inquiry process. Typical responses during the *discovery* phase with families, and during the semi-structured interviews with the adolescents later, were the following: ‘I was not aware that my family feels this way’ (A1, female, 11 years old; A2, female, 12 years old); ‘I did not know they care for me so much’ (A5, male, 13 years old; A8, male, 12 years old); ‘Was not aware how my son felt’ (F7, male, 1 years old). For some adolescents it was difficult to hear positive comments, stories and memories surrounding them, as they appeared to be shy (A2, A6). One cried (A5) and one (A1) placed her hands over her ears because she experienced the attention as overwhelming. Some adolescents were able to express their feelings verbally, or non-verbally, by hugs, sitting closer to a family member or sitting on the lap of a particular family member who made positive comments. Cooperrider et al. (2008) referred to this phase as discovering, valuing and appreciating all factors that give ‘life to a living system’ and Cooperrider and Whitney (2005) as a meaning-making and reflecting phase (cf. Anderson & Sabatelli 2011).

### Values questions

These questions supported the BarOn EQ-i:YV questions in the interpersonal and intrapersonal constructs, focusing on perceptions of self and others. Family members valued the intellectually challenged adolescent’s sense of self, referring to several domains such as their relationship with the adolescent, his or her attributes, abilities and disabilities, which they viewed as the adolescent’s self. Families acknowledged the adolescent’s uniqueness, friendliness, kindness, helpfulness, shyness, always trying his or her best, being a good swimmer, singer or cricket player, and showing perseverance and endurance despite physical and intellectual disabilities. This naming of selves (e.g. a kind self or a shy self), according to Polster (2005), is an important process of self-formation, as it gives the self a brightness and recognisability and has implications for likely behaviour and feelings. By listening to families’ stories, the intellectually challenged adolescents became aware of how others saw them and they introjected new perceptions about their selves (if they were not aware of these perceptions) and altered old perceptions and experiences they had of their selves. These experiences according to Young and Colin (2004) take place through social processes, such as everyday interactions between people and conversations, as they unfold, and according to Hutchinson (2003), in relationships with others (Yontef 1997). How the intellectually challenged adolescent perceives himself or herself does not take place in isolation but is socially constructed and reconstructed through realities, external social influences and dynamic forces of the field (Gergen 2011).

#### Core life-giving factor or value questions

These questions formed part of the appreciative inquiry process where families were made aware of family values, their own uniqueness and experiences of each other. Some typical responses were: ‘Family means everything’ (A2, female, 12 years old); ‘Families stay together, support and protect each other’ (A10, male, 13 years old); ‘Family roots are important: where you came from, who you are, where you are going’ (A4, female, 12 years old).

These responses emphasised the central role that families play in the development of adolescents’ identities and in the formation of close relationships through support and encouragement. These findings are in line with those of prior studies by Anderson and Sabatelli (2011), Becvar and Becvar (2013), Fomby and Osborn (2010) and Silkos and Kerns (2006). They further illustrated some degree of emotional bonding, a shared sense of history and expectations from family members. Cooperrider Dole et al. (2008) point out the importance of having a safe and mutually supportive family environment, where family members can care for one another and positively influence each other’s development.

#### Challenges and conflict within families

Conflict arises when something is really important to us and is not seen as important or valuable by others, and conflict is intersubjective within relationships. In conflict situations, energy is released that can produce change and become a transformative source within the family and within the individual himself or herself. According to Cooperrider et al. (2008), ‘negative’ comments during meetings or in data can be managed, redirected and put into affirmatives. Negative comments and conflict situations were mentioned by family members during the intervention meetings, such as not being heard by family members (A1, A2), or sibling rivalry (F3, F12), or not being respected, being called ‘stupid’ or ‘retarded’ (A8). These comments were rephrased during the family meetings. Small steps were put in place during the *designing* phase to show how the adolescents can be heard, respected and valued.

#### Dream phase (three wishes)

These were different for each family unit, as some families (F5, F6) focused on more concrete desires such as living in better conditions, being more financially secure and being able to support the adolescent more, with extra activities or therapy, so the person can reach his or her full potential. Families envisioned their intellectually challenged adolescent as more responsible (A2) and self-supportive (A11) when he or she grows older. Families dreamed of fewer fights and less conflict between siblings and the intellectually challenged adolescent (A3, A8) and of being seen as a ‘normal family’ (F10, F12). The dreams of the intellectually challenged adolescents focused on their desire to have a better and closer relationship with their parents (especially in absent father and mother households [F6, F8] and in strained relationships with parent[s] [A7] and siblings). Typical responses of adolescents mentioned a desire to be accepted by their siblings and peers, to be heard (have a voice), to be seen for ‘who they are’ and not focusing on what they cannot do (A6). According to Cooperrider and Whitney (2005), our dreams, visions and positive images bring to the fore more positive actions, altering our future (anticipatory principle) by creating inspiring new images of what we want. This then allows us to make small changes in the present (Kelm 2005).

The *design* phase is a co-constructing phase, a planning phase where families’ wishes become a reality, by crafting provocative propositions, questions and visions for the future (Cooperrider et al. 2008). The questions that we ask set the stage for what we find. What we discover becomes the stories out of which the future is conceived and constructed (Cooperrider & Whitney 2005). The propositions were different for each family unit. Through brainstorming and dialogue, members were able to contribute to practical steps for *how* their dreams of being heard, being respected, validated and accepted by family members and society, as well as of spending more quality time together, could become a reality. Some of the families’ responses were: ‘We are a growing family – encouraging each other to discover own potential, bringing out the best of the individual and family’ (A12, male, 12 years old); ‘We are a family that stands together, showing courage to share with others, to enable us to grow’ (A3, female, 14 years old); ‘We are a family with a vision for all our children – including our disabled child’ (A2, female, 12 years old).

According to the social identity theory’s perspective, individuals tend to categorise themselves by firstly comparing themselves to and then evaluating other groups. This social comparison is based on the characteristics of the group, its members and benefits. The outcome of such social comparisons, and the values placed on the group and on the individual, largely determine our social identity (Festinger 1954; Stets & Burke 2000). The majority of families expressed a desire to belong to and to be accepted by a social group without being judged for raising an intellectually challenged adolescent. During the *designing* phase, family members decided as part of their family plans to reach out to other family members with similar challenges, in order to get support and understanding and to address their own family difficulties (F6, F10).

Questions raised during the *design* phase were the following: how do we embrace our challenges? How do we respect each other’s privacy and opinions? How do we communicate with each other in ways besides shouting and screaming? What is our vision for our family and for our intellectually challenged adolescent? These questions assisted in moving the system to positive action and intended results. This phase involved the collective construction of positive images of the family’s future. It underlines the social constructionist perspective, which states that all experiences are subjective and that human beings recreate and reconstruct themselves through an ongoing, never static *process*. This construction is embedded within relationships with others, which cannot be separated from our social-cultural and historical context (Gergen 2011). Practical designs from family members were parents’ changing work rosters and shifts in order to spend more time with the family and adolescent (F4), enhancing communication by making constructive efforts such as having meals together (F10, F8), making use of a communication board (F12, F3) with important dates (birthdays, school functions) and the allocation of each member’s chores. Parents recognised the importance of ‘letting go’ by allowing adolescents to go on school trips and inviting and socialising with friends at their homes. This will enhance the independent and autonomous selves of the intellectually challenged adolescents (A7, A1, and A2).

During the *destiny phase*, also called the *delivery phase*, dreams become a reality through continuous dialogue (Gergen & Gergen 2004). It is where families shared positive images in co-creating the future. This phase was different for each family, as their dreams, challenges and plans were unique. This phase focused on members’ commitment to what needed to be done, implementing the designing plans in order to support and strengthen relationships. Questions put to families during this phase stimulated dialogue and integrated the whole intervention process. Appreciative inquiry dialogue, according to Cooperrider et al. (2008), creates guiding *images* for the future, which in turn evoke positive emotions and move people toward a choice for *positive action* (Cooperrider 1999). It is argued that all human systems display a continuing or ongoing ‘inner newsreel’ that is best understood through the concept of inner dialogue. *Inner dialogue* functions as an inner dialectic (interaction) between positive and negative adaptive statements (Cooperrider et al. 2008). Typical comments by families included the following: ‘The meeting was meaningful’ (A9, male, 13 years old); ‘It was enlightening – gaining insight into my family’s feelings’ (A3, female, 14 years old) and ‘adolescent’s feelings’ (A1, female, 11 years old); ‘[*it*] helped us to address difficult issues’ (A8, male, 12 years old).

Dialogue, expression and positive emotions are at the core of appreciative inquiry and underline the positive principle. Positive expression not only broadens thinking and action, but it undoes negative ones; it builds resiliency and creates upward spirals – for possibilities and change (Cooperrider & Whitney 2005).

During the family interventions, strengths, weaknesses, opportunities and threats, internal and external, were noted. General *strengths* were strong religious and cultural beliefs (see family quotes), positive attitudes in embracing the challenges of having to take care of an intellectually challenged adolescent, strong bonds, relationships and support. *Weaknesses* or challenges were being overprotective, not including the adolescent in family decisions (A4, A8) and a sense of guilt in parents, who blamed themselves for the diagnosis of the intellectually challenged adolescent (A7). These feelings of shame from siblings and family members (F8, F10) led to close system family functioning, not allowing for changes and natural feedback processes. Other factors contributing to this were a lack of insight into the positive possibilities in raising an intellectually challenged child, single-parent family structures and financial difficulties (F5, F6). Families with a positive outlook on life focused on *opportunities* such as sharing their experiences and challenges with friends (F3, F11) or using programmes available not only for the adolescent but for the family as a whole, by attending school programmes and requesting help and assistance (F2, F4). Families identified *threats* as judgemental behaviour from society that has a negative impact on how they viewed themselves, stigmatisation and name calling of the intellectually challenged adolescent (A6, A9), which influenced their own perceptions and behaviour towards the adolescent (F10, A7). Parents and siblings voiced feelings of insecurity and fears regarding what was going to happen when the adolescent grows up and who is going to take care of him or her.

## Limitations

One of the limitations in this study may be that it would have been ideal to do follow-ups with the families of the experimental group regarding the implementation of the collective family plans that were constructed (verbally) during the *designing* phase. In terms of semi-structured interviews with the adolescents, it would have been ideal to have a secondary follow-up to determine whether the implementation of the families’ collective plans (*destiny* phase) had altered their perception of their self (intrapersonal) and that of others (interpersonal).

## Recommendations

In this study, families participated as 12 individual family units in the appreciative inquiry intervention, but it is recommended that in future research, the approach be applied on a larger scale, where a population of families with intellectually challenged adolescents be grouped together. The development of an appreciative inquiry integrative coaching model is suggested, to address challenges in the family environment, relationship forming and with the formation and strengthening of the intellectually challenged adolescent’s sense of self.

Findings on the FES can serve as a motivation to conduct future research in supporting and coaching families with intellectually challenged adolescents. On the BarOn EQ-i:YV no norms for children and adolescents with intellectual and developmental disabilities were available to enable comparisons in that cohort. This can be explored by researchers, to develop an internal reliability of the BarOn EQ-i:YV on the different scale items (questions) and generate norms for children and adolescents with intellectual and developmental disabilities.

## Conclusion

The purpose of this study was to explore and describe how the sense of self is viewed by intellectually challenged adolescents and their families and how it could be strengthened within familial relationships by using appreciative inquiry. The findings were consistent with previous research regarding the vital role that families play in child development, the forming of close bonds and relationships, socialising and the construction of an identity (Becvar & Becvar 2013; Erikson 1968). However, previous research was largely based on a deficit model that focused on the limitations of and symptomatic behaviour by intellectually challenged adolescents. Strengthening the sense of self of intellectually challenged adolescents involves the whole family, which needs to make time for conversations, caring, seeing the best in each other and to affirm their uniqueness. Strengthening came from valuing, listening, collaborating and showing respect to the adolescents. In this study, the appreciative inquiry family intervention created a platform for intellectually challenged adolescents to see themselves through the eyes of their family members, affirming and/or altering their own perceptions of self. Cottle (2003) suggests that this kind of affirmation takes place through our *relatedness* with others, discovering another person (otherness) out there apart from us, and with whom we form a kinship (relationship) or identification with – *a rootedness*.

The quantitative findings indicated no statistically significant changes after the intervention, allowing the conclusion that adolescents’ sense of self was strengthened within the familial relationships by using appreciative inquiry as an intervention technique. This could be a result of the small sample size and of the BarOn EQ-i:YV having standardised norms only for normal adolescents and not for intellectually challenged adolescents. Results therefore had to be compared with the norms of normal adolescents.

Moreover, the FES questionnaire was completed by all family members at home and not in a controlled environment. It was clearly indicated that the FES questionnaire was to be completed individually, but it is unknown whether any parental influence played a role in the findings. Further results on the FES findings (*cohesion* and *conflict*) comparing the scores with ‘normal’ families raising typically developing adolescents to the scores of families with intellectually challenged adolescents show that the families of intellectually challenged adolescents can be considered as families in distress. This could be a result of the unique challenges that families have to face in living with and raising intellectually challenged adolescents.

The qualitative data obtained from the appreciative inquiry intervention approach showed potential for strengthening intellectually challenged adolescents’ sense of self within familial relationships. These findings were evident in the close emotional bonds and familial relationships, togetherness and feelings of belonging observed during the intervention but, most importantly, the role that the family played in validating and accepting the adolescents for who they are. Despite families being aware of the adolescents’ disability and the stigma attached to the disability, the majority of families were able to experience the sense of self of the intellectually challenged adolescents positively, and not as a ‘disabled’ self.
